# Real-Time Non-invasive Assessment of Cerebral Hemodynamics With Diffuse Optical Spectroscopies in a Neuro Intensive Care Unit: An Observational Case Study

**DOI:** 10.3389/fmed.2020.00147

**Published:** 2020-04-28

**Authors:** Rodrigo M. Forti, Marilise Katsurayama, Julien Menko, Lenise Valler, Andres Quiroga, Antonio L. E. Falcão, Li M. Li, Rickson C. Mesquita

**Affiliations:** ^1^Institute of Physics, University of Campinas, Campinas, Brazil; ^2^Brazilian Institute of Neuroscience and Neurotechnology, Campinas, Brazil; ^3^Clinical Hospital, University of Campinas, Campinas, Brazil; ^4^Department of Emergency Medicine, Albert Einstein College of Medicine, Bronx, NY, United States; ^5^School of Medical Sciences, University of Campinas, Campinas, Brazil

**Keywords:** cerebral blood flow (CBF), neuro intensive care unit, diffuse correlation spectroscopy (DCS), diffuse optical spectroscopy (DOS), aneurysmal subarachnoid hemorrhage

## Abstract

Prevention of secondary damage is an important goal in the treatment of severe neurological conditions, such as major head trauma or stroke. However, there is currently a lack of non-invasive methods for monitoring cerebral physiology. Diffuse optical methods have been proposed as an inexpensive, non-invasive bedside monitor capable of providing neurophysiology information in neurocritical patients. However, the reliability of the technique to provide accurate longitudinal measurement during the clinical evolution of a patient remains largely unaddressed. Here, we report on the translation of a hybrid diffuse optical system combining frequency domain diffuse optical spectroscopy (FD-DOS) and diffuse correlation spectroscopy (DCS) for real-time monitoring of cerebral physiology in a neuro intensive care unit (neuro-ICU). More specifically, we present a case study of a patient admitted with a high-grade aneurysmal subarachnoid hemorrhage, who was monitored throughout hospitalization. We show that the neurophysiological parameters measured by diffuse optics at the bedside are consistent with the clinical evolution of the patient at all the different stages following its brain lesion. These data provide support for clinical translation of DOS/DCS as a useful biomarker of neurophysiology in the neuro-ICU, particularly in locations where other clinical resources are limited.

## Introduction

One of the main goals in the care of patients with severe neurological conditions, such as a major head trauma or stroke, is to prevent secondary damage. Additional insults caused by primary damage affect and may be affected by both the systemic and cerebral physiologies (1–4). To this end, close patient monitoring following acute brain lesions is essential to prevent secondary damage, and it may directly improve mortality and morbidity rates (5–7). Yet, monitoring of neurocritical patients has been limited to intermittent systemic physiological monitors and/or snapshot measurements through computed tomography (CT) or magnetic resonance imaging (MRI) (8–11). Continuous cerebral monitoring is expensive, and it commonly requires invasive catheters, which limits its use to few, more severe cases, and it is seldom used in low-budget environments (8).

Among the few potential non-invasive techniques to probe cerebral physiology at the bedside, Transcranial Doppler ultrasound (TCD) is generally used to monitor cerebral blood flow velocity (CBF_v_) in the large arteries (12). However, TCD is operator-dependent, not suitable for long-term monitoring, and is not sensitive to changes at the microvasculature. In addition, ~10% of the subjects do not have appropriate acoustic windows (13, 14). Electroencephalography (EEG) is another commonly used non-invasive technique to assess electrophysiological activity of the brain. However, transcranial EEG has poor spatial resolution and is very sensitive to artifacts (15).

More recently, diffuse optical methods have been proposed as a non-invasive and continuous bedside monitor capable of providing neurophysiological information in neurocritical patients (16–31). By shining near-infrared light from the scalp, diffuse optics can measure microvascular CBF and cerebral hemoglobin concentrations (32–38). In addition, the combination of blood flow and oxygenation provides an estimate of the cerebral metabolic rate of oxygen (CMRO_2_) (39, 40). In its simplest form, near-infrared spectroscopy (NIRS) is a diffuse optical technique currently employed as a complementary physiological monitor in several clinical scenarios (23, 25, 29, 41–45). However, since NIRS is only capable of measuring *changes* in oxygenation, it has been limited as trend monitors, therefore being susceptible to systemic physiological oscillations and diminishing its usefulness for longitudinal monitoring of patients (46–51).

Some studies have employed frequency- (FD-DOS) and time-domain (TD-DOS) diffuse optical spectroscopy to measure absolute tissue oxygenation. Either FD-DOS or TD-DOS can be combined with diffuse correlation spectroscopy (DCS) for measurements of CBF and metabolism. In particular, DCS-based measurements of have been previously validated against several gold standard CBF measures (37). The combination of DOS and DCS has been explored to monitor patients in several clinical scenarios, e.g., for neonatal monitoring (34, 52–55), during cerebrovascular interventions (18, 21, 22, 27, 28, 56), as well as for monitoring of neurocritical patients (16, 17, 31). However, these studies mostly focused on the effects of specific events and interventions. Concerning the practical clinical application of diffuse optics, the important question regarding the relationship between neurophysiology, as measured by DCS/DOS, and the clinical evolution of patients during hospitalization remains largely unaddressed.

## Case Description

We describe the case of a 62-year-old female patient who was admitted to the neuro intensive care unit (neuro-ICU) following aneurysmal subarachnoid hemorrhage (aSAH). She was an obese smoker with inconsistent adherence to her anti-hypertensive regimen. The patient was recruited during admission to the neuro-ICU of the Clinical Hospital at the University of Campinas, Brazil.

The patient was admitted after a severe headache for 3 days associated with nausea, vomiting and elevated blood pressure. She rapidly progressed to coma right after admission. The first CT scan after admission revealed a right middle cerebral artery (MCA) aSAH with Grade V on the Hunt & Hess scale and Grade III on the Fisher Scale. The TCD measurement performed on the first day of hospitalization did not show any signs of vasospasm (mean CBFv of 73 and 83 cm/s, for the right and left MCA, respectively). In the end of the first day after hospitalization the patient underwent an emergency ventriculostomy with EVD placement due to high intracranial pressure.

The CT scan obtained in the third and fourth days after admission revealed a diffuse cerebral edema with collapse of lateral ventricles, as well as an extensive ischemic area on the ipsilesional hemisphere. The TCD performed in the fourth day found mean CBFv of 64.2 and 68.3 cm/s on the right and left MCAs, respectively. In the same day, the patient evolved with non-photo reacting pupils and absent corneal reflex. The clinical examination remained unchanged even after analgesic and sedative drug withdrawal. With this prognostic, the clinical staff opted for comfort care. The patient died on the ninth day after hospitalization.

## Optical Assessment

### Experimental Protocol

The experimental protocol was designed so that the optical measurements would not interfere with any clinical practices. Therefore, the timing of each optical monitoring session was purposely chosen to avoid conflicts with any planned clinical intervention. Most of the monitoring sessions happened in the early afternoon, prior to family visitation hours. No treatment decisions were made based on the optical measurements.

The optical system used in this study consists of a hybrid instrument employing a DCS module and a DOS module (Supplementary Figure 1). For details about the optical instrumentation and the optical probe, see Supplementary Methods. In this work, we opted to focus on a single DCS source-detector separation (2.5 cm) and two DOS wavelengths (690 and 846 nm). These separations allowed the optical device to probe the external surface of the prefrontal cortex (PFC), as it was shown in previous studies (19, 38). We developed a user-friendly graphical user interface (GUI) based on current clinical monitors to control the operation of each module and to display in *real-time* CBF, OEF, and CMRO_2_.

We utilized the FD-DOS data to estimate the absolute oxy- and deoxy-hemoglobin concentrations (HbO and HbR, respectively). Total hemoglobin concentration was calculated as the sum of HbO and HbR (i.e., HbT = HbO + HbR). Tissue oxygen saturation (StO_2_) was calculated as the fraction of HbO considering all hemoglobin concentrations (i.e., StO_2_ = HbO/HbT). Oxygen extraction fraction (OEF) was computed as OEF = (SaO_2_ − StO_2_)/(0.75^*^SaO_2_), where SaO_2_ is the arterial blood oxygen saturation (39, 55). DOS data was combined with DCS data to provide cerebral blood flow (CBF), and metabolism. From CBF and OEF, we estimated the cerebral metabolic rate of oxygen (CMRO_2_) as CMRO_2_ = Hgb ^*^ CBF ^*^ OEF (39, 40, 55). All neurophysiological parameters were calculated for each time point continuously every 6 s. The whole analysis procedure is detailed in the Supplementary Methods and can be found elsewhere (32, 33, 57).

Optical measurements were taken for five of the 9 days of the patient hospitalization for at least 1 h per day. The optical probe was held on the forehead with an elastic strap. Each brain hemisphere was measured sequentially, for at least 30 min on each side. All monitoring sessions were supervised by a specialized neurologist. Mean arterial pressure (MAP), arterial blood saturation (SaO_2_), hemoglobin concentration (Hgb) and CBF_v_ from TCD were retrospectively recovered from the medical records and were taken at most an hour before or after the optical measurements.

Due to the lack of continuous measurement of systemic physiology, we opted to report the average data points of each neurophysiological parameter calculated across each optical monitoring session. Supplementary Figure 2 shows the continuous measurement of all the optical-derived parameters for one representative monitoring session.

### Optical Monitoring

**Figure 1:**
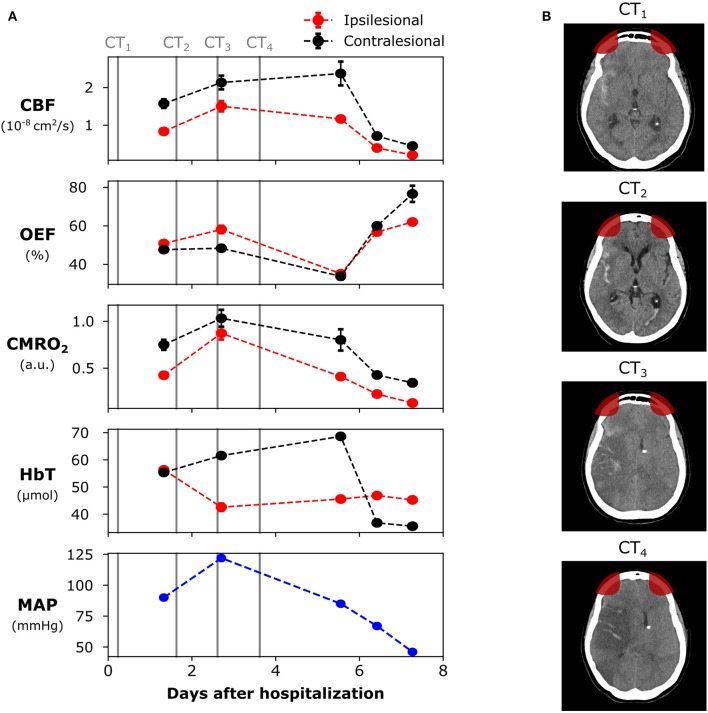
Evolution of the brain lesion in a 62 years old female patient following a high-grade aneurysmal subarachnoid hemorrhage (aSAH). **(A)** Neurophysiological parameters measured with the diffuse optical system, as well as the systemic mean arterial pressure (MAP). **(B)** Computed tomography (CT) images at different days during hospitalization (marked as vertical lines in the left panel). The patient died 9 days after hospitalization. The red areas in the CT images represent the optical sensitivity region. The error bars of each point represent the standard deviation of each parameter across the monitoring time-window. For some days, the standard deviation was too small to be shown. CBF, cerebral blood flow; OEF, oxygen extraction fraction; CMRO_2_, cerebral metabolic rate of oxygen; HbT, total hemoglobin concentration.

Figure 1 shows a timeline of the main events during the patient hospitalization, along with the daily averages of all the cerebral measurements obtained with our diffuse optical system along with MAP and the CT scans performed during the whole hospitalization period.

We started monitoring the patient with our diffuse optical system on the second day following admission, ~32 h after hospitalization. At that time, MAP was 90 mmHg and we found lower CBF and CMRO_2_ on the ipsilesional side of the PFC when compared to the contralesional side, consistent with lower metabolism following a brain hemorrhage. The OEF and the cerebral blood volume (CBV, as estimated by HbT) were similar between the two hemispheres

On the third day of hospitalization, MAP increased to 122 mmHg, and we observed an overall increase in CBF and CMRO_2_. The increase in CBF and CMRO_2_ was comparatively higher in the ipsilesional side. In addition, an increase in ipsilesional OEF and a decrease in ipsilesional HbT were also measured, suggesting high metabolic demand due to low blood availability in tissue. No optical monitoring was taken between the fourth and the fifth days due to intermittent clinical care to assist the patient.

On the sixth day of hospitalization, OEF was below 40% in both hemispheres. Compared to the second day of hospitalization, CBF increased by 40 and 51% in the ipsilesional and contralesional sides, respectively. However, the increase in CBF was not followed by any significant changes in CMRO_2_ (Figure 1). From the second to the sixth day after hospitalization, HbT increased in the contralesional side and decreased in the ipsilesional side. MAP at the sixth day was 85 mmHg. For the following 2 days, ipsilesional CBF and CMRO_2_ further decreased, respectively, by 82 and 69% (compared to day 6), and OEF dramatically increased by ~76%. CBV (as estimated by HbT) decreased in the contralesional side, reaching the levels of the ipsilesional side. The ipsilesional CBV remained relatively stable between the second and the ninth days of hospitalization. MAP decreased to 67 and 45 mmHg during the last two monitoring sessions, respectively.

## Discussion

Diffuse optics has been previously validated in medical settings as an alternative bedside monitoring tool, and it holds potential to personalize clinical management. Despite limitations in the penetration depth inherent to any diffuse optical technique (32, 37, 38), both DOS and DCS are capable of monitoring cerebral hemodynamics non-invasively and in *real-time*, without the use of exogenous contrasts, ionizing radiation, and without interfering with current clinical practices. Unlike global systemic physiological monitors, the principles of light propagation limit the measurement to a region of interest, which can be useful to monitor specific regions, such as an ischemic penumbra. In addition, due to its portability and low cost, diffuse optical methods can be very useful to clinicians in places where clinical care conditions are restricted, such as in military fields and in developing regions. Even in better clinical conditions, access to daily neuroimaging data for longer periods of time may not be readily available.

Here, we report the results from an observational case report concerning a patient severely affected by aSAH, who was admitted almost 3 days after the event. For simplicity, we limited our measurements to the forehead, and thus we were only sensitive to focal *changes* in the PFC, which was sufficient to elucidate the main events during the evolution of the patient's brain injury. Since we focused on the feasibility of performing longitudinal measurements in the neuro-ICU, we minimized both the size of the optical probe and the monitoring time per day to reduce the interference with clinical interventions. However, we note that these choices are not intrinsic limitations of the optical system. The optical probes are small and customized so that they can be temporarily detached from patients for any necessary reason. Since our optical system measures *absolute* values, we are not limited to a baseline or reference period. Also, the system's high portability allows its relocation alongside the patient with minimal time and effort. Additionally, *long-term* continuous monitoring (i.e., several hours per day) poses no extra-challenge in the present system. In fact, previous studies have reported diffuse optical measurements at the ICU for as long as 8 h per day, both in adults (16, 31) and in neonates (58).

The results from this pilot observational case study provide evidence that neurophysiological information derived from DOS and DCS is consistent with the triphasic evolution of SAH: hypoperfusion, hyperemia, and vasospasm (59, 60). During the first day of monitoring, the patient had poor ipsilesional CBF and CMRO_2_ but normal OEF when compared to the contralesional side, consistent with a hypoperfusion phase. In a previous PET study, Yundt et al. found similar trends of reduced CBF and CMRO_2_, but unaffected OEF, during the early phases of SAH (61, 62). This pattern of low CBF and CMRO_2_ but unaffected OEF may be related to an ongoing ischemia, to the start of the vasospasm phase or to a sign that the injury-related neurologic deficits have not yet peaked. Based on the admission CT scan (Figure 1), our results are most likely related to an ongoing ischemia. The initial SAH insult may impact mitochondrial function and diminish oxidative metabolism in favor of anaerobic metabolism (63), which may further explain our findings of low CBF and CMRO_2_, but unaffected OEF.

At the third day after hospitalization, we found an increase in OEF at the ipsilesional side, which was associated with a bilateral increase in CBF and CMRO_2_ (with a comparatively higher ipsilesional increase). Previously, Yokose et al. found decreased ipsilesional oxygen saturation (which corresponds to increased OEF) in severe SAH patients with angiographic vasospasm with TD-DOS (64). Thus, the higher OEF during the third day after hospitalization could be an indicative of vasospasm. The fact that the patient evolved with an ipsilesional ischemia between the fourth and fifth days is another indicative of vasospasm. Taken together, our results corroborate Yokose et al.'s (64), and reinforces the argument that decreases in tissue oxygenation (or, similarly, increases in OEF) may be a more sensitive measure of vasospasm than CBF_v_ measured by TCD. Of note, prolonged elevation of ICP may also induce ischemia in aSAH patients; in this study we did not assess ICP, therefore we cannot confirm the real cause for ischemia in this patient.

We also measured increased CBF and decreased OEF in the contralesional side of the patient between the second and the sixth days following hospitalization, with no significant difference in MAP. The increase in CBF with lower OEF is an indicative of cerebral hyperemia that may have been secondary to an inflammatory mediated process causing vasodilation of the microvascular bed (63, 65). The cerebral hyperemia in the contralesional side may also be explained by an attempt to restore metabolic balance in the affected tissue. However, by this time the patient had already developed a large ischemic insult, and only the healthy tissue was capable of increasing CBF and appropriately oxygenating the tissue. Additionally, the ipsilesional ischemia may explain the fact that CBV (as measured by HbT) remained approximately constant across the different monitoring days (besides the first monitoring session).

Overall, the optically derived hemodynamic parameters were sensitive to the mismatch between the physiology of each hemisphere. We note, though, that further clinical studies with larger populations are required to assess the accuracy of the technique to find specific events, and/or to measure the effects of potential treatments. Together, our results reinforce that diffuse optics may provide useful information at the bedside, which could aid clinicians to make decisions based on each patient's neurophysiology. The *real-time* access to patient's neurophysiological condition may positively impact clinical care and patient outcome (5–7). In addition, our results show the feasibility of performing diffuse optical monitoring in a restricted environment in a developing country, where access to other methods of cerebral monitoring are not always readily available.

Even though the optical results show strong agreement with clinical outcome, it is worth noting that the optical parameters can be more accurate. In this work, we modeled the head as a semi-infinite medium for the DCS/DOS data analysis. By using the simplest model for analysis in order to get real-time monitoring, our results do not account for extracortical contributions. Thus, it is likely that we have underestimated the cerebral physiology *changes* (33, 37, 38, 66). In the future, multi-layer models can be used to isolate extracortical contributions from the optical signal (67–71). However, these models either require an extra step in the monitoring setup or may not yet be reliable for clinical use. Nonetheless, the quantification of the neurophysiological changes does not affect the comparison between hemispheres, since extracerebral contributions are expected to similarly affect both hemispheres. Last, the optical-derived CBF has physical units (i.e., cm^2^/s) rather than the more usual clinical units (i.e., ml/100 g of tissue/min). Some authors have proposed the use of an indocyanine-green (ICG) bolus to recover absolute CBF from the optical signal, which may be used to convert the continuous CBF index from DCS to the absolute clinical units (72–74).

## Conclusion

To summarize, in this study we presented an observational case report recorded during the translation of a hybrid diffuse optical system for real-time monitoring of cerebral hemodynamics inside a neuro-ICU. Our results suggest that optics may provide supporting information to monitor secondary damage following brain injury, and that optics can be safely and non-invasively applied early during hospitalization, at the bedside and with minimal interference with standard clinical practices. The optically-derived hemodynamic parameters provide neurophysiological information at the microvasculature, which was consistent with the clinical outcome. Although we focused on one single case, our results support that diffuse optics can be a reliable tool for bedside monitoring and encourage further clinical validations with DCS/DOS in the neuro-ICU. Translation of diffuse optics to clinical settings can positively impact patient outcome and can be especially useful in low-budget hospitals and in remote areas, where cerebral physiology monitoring is essential but not readily available.

## Data Availability Statement

The datasets generated for this study are available on request to the corresponding author.

## Ethics Statement

The studies involving human participants were reviewed and approved by Ethics Committee of the University of Campinas (Comitê de Ética em Pesquisa). The patients/participants provided their written informed consent to participate in this study. Written informed consent was obtained from the individual(s) for the publication of any potentially identifiable images or data included in this article.

## Author Contributions

AQ, LV, MK, RF, and RM collected all the data. RF wrote the first draft of the manuscript and conducted the final analysis. RF and RM designed the data analysis protocol. All authors assisted with data interpretation, contributed to manuscript revision, read, and approved the submitted version.

## Conflict of Interest

The authors declare that the research was conducted in the absence of any commercial or financial relationships that could be construed as a potential conflict of interest.

## References

[1] Al-Tamimi YZ Orsi NM Quinn AC Homer-Vanniasinkam S Ross SA. A review of delayed ischemic neurologic deficit following aneurysmal subarachnoid hemorrhage: historical overview, current treatment, and pathophysiology. World Neurosurg. (2010) 73:654–67. 10.1016/j.wneu.2010.02.005 20934153

[2] Cunningham AS Salvador R Coles JP Chatfield DA Bradley PG Johnston AJ. Physiological thresholds for irreversible tissue damage in contusional regions following traumatic brain injury. Brain. (2005) 128:1931–42. 10.1093/brain/awh536 15888537

[3] Vergouwen MDI Vermeulen M van Gijn J Rinkel GJE Wijdicks EF Muizelaar JP. Definition of delayed cerebral ischemia after aneurysmal subarachnoid hemorrhage as an outcome event in clinical trials and observational studies. Stroke. (2010) 41:2391–5. 10.1161/STROKEAHA.110.589275 20798370

[4] Werner C Engelhard K. Pathophysiology of traumatic brain injury. Br J Anaesth. (2007) 99:4–9. 10.1093/bja/aem131 17573392

[5] Papanikolaou J Makris D Karakitsos D Saranteas T Karabinis A Kostopanagiotou G. Cardiac and central vascular functional alterations in the acute phase of aneurysmal subarachnoid hemorrhage. Crit Care Med. (2012) 40:223–32. 10.1097/CCM.0b013e31822e9fab 21926590

[6] Sarrafzadeh AS Vajkoczy P Bijlenga P Schaller K. Monitoring in neurointensive care – the challenge to detect delayed cerebral ischemia in high-grade aneurysmal SAH. Front Neurol. (2014) 5:8–11. 10.3389/fneur.2014.00134 25101052 PMC4104636

[7] Messerer M Daniel RT Oddo M. Neuromonitoring after major neurosurgical procedures. Minerva Anestesiol. (2012) 78:810–22. 22561676

[8] Le Roux P Menon DK Citerio G Vespa P Bader MK Brophy GM. Consensus summary statement of the international multidisciplinary consensus conference on multimodality monitoring in neurocritical care. Intensive Care Med. (2014) 40:1189–209. 10.1007/s00134-014-3369-6 25138226

[9] Sandsmark DK Kumar MA Park S Levine JM. Multimodal monitoring in subarachnoid hemorrhage. Stroke. (2012) 43:1440–5. 10.1161/STROKEAHA.111.639906 22426466

[10] Oddo M Villa F Citerio G. Brain multimodality monitoring. Curr Opin Crit Care. (2012) 18:111–8. 10.1097/MCC.0b013e32835132a5 22322259

[11] Roh D Park S. Brain multimodality monitoring: updated perspectives. Curr Neurol Neurosci Rep. (2016) 16:56. 10.1007/s11910-016-0659-0 27095434 PMC4863980

[12] Bishop CCR Powell S Rutt D Browse NL. Transcranial Doppler measurement of middle cerebral artery blood flow velocity: a validation study. Stroke. (1986) 17:913–5. 10.1161/01.STR.17.5.913 3764963

[13] Purkayastha S Sorond F. Transcranial Doppler ultrasound: technique and application. Semin Neurol. (2013) 32:411–20. 10.1055/s-0032-1331812 23361485 PMC3902805

[14] Demchuk AM Christou I Wein TH Felberg RA Malkoff M Grotta JC. Accuracy and criteria for localizing arterial occlusion with transcranial Doppler. J Neuroimaging. (2000) 10:1–2. 10.1111/jon20001011 10666975

[15] Banu SH EEG in ICU: a monitoring tool for critically ill patient. Bangladesh Crit Care J. (2014) 2:28–34. 10.3329/bccj.v2i1.19954

[16] Baker WB Balu R He L Kavuri VC Busch DR Amendolia O. Continuous non-invasive optical monitoring of cerebral blood flow and oxidative metabolism after acute brain injury. J Cereb Blood Flow Metab. (2019) 39:1469–85. 10.1177/0271678X19846657 31088234 PMC6681541

[17] Busch DR Balu R Baker WB Guo W He L Diop M Lawrence K. Detection of brain hypoxia based on noninvasive optical monitoring of cerebral blood flow with diffuse correlation spectroscopy. Neurocrit Care. (2019) 30:72–80. 10.1007/s12028-018-0573-1 30030667 PMC6528475

[18] Forti RM Favilla CG Cochran JM Baker WB Detre JA Kasner SE. Transcranial optical monitoring of cerebral hemodynamics in acute stroke patients during mechanical thrombectomy. J Stroke Cerebrovasc Dis. (2019) 28:1483–94. 10.1016/j.jstrokecerebrovasdis.2019.03.019 30975462 PMC6686873

[19] Kim MN Durduran T Frangos S Edlow BL Buckley EM Moss HE. Noninvasive measurement of cerebral blood flow and blood oxygenation using near-infrared and diffuse correlation spectroscopies in critically brain-injured adults. Neurocrit Care. (2010) 12:173–80. 10.1007/s12028-009-9305-x 19908166 PMC2844468

[20] Delgado-Mederos R Gregori-Pla C Zirak P Blanco I Dinia L Marín R. Transcranial diffuse optical assessment of the microvascular reperfusion after thrombolysis for acute ischemic stroke. Biomed Opt Express. (2018) 9:1262. 10.1364/BOE.9.001262 29541519 PMC5846529

[21] Gregori-Pla C Blanco I Camps-Renom P Zirak P Serra I Cotta G. Early microvascular cerebral blood flow response to head-of-bed elevation is related to outcome in acute ischemic stroke. J Neurol. (2019) 266:990–7. 10.1007/s00415-019-09226-y 30739181

[22] Favilla CG Forti RM Zamzam A Detre JA Mullen MT Yodh AG. Perfusion enhancement with respiratory impedance after stroke (PERI-Stroke). Neurotherapeutics. (2019) 16:1296–303. 10.1007/s13311-019-00744-1 31140115 PMC6985403

[23] Obrig H. NIRS in clinical neurology — a ‘promising’ tool? Neuroimage. (2014) 85:535–46. 10.1016/j.neuroimage.2013.03.045 23558099

[24] Buckley EM Parthasarathy AB Grant PE Yodh AG Franceschini MA. Diffuse correlation spectroscopy for measurement of cerebral blood flow: future prospects. Neurophotonics. (2014) 1:011009. 10.1117/1.NPh.1.1.011009 25593978 PMC4292799

[25] Ritzenthaler T Cho T-H Mechtouff L Ong E Turjman F Robinson P. Cerebral near-infrared spectroscopy a potential approach for thrombectomy monitoring. Stroke. (2017) 48:3390–2. 10.1161/STROKEAHA.117.019176 29089454

[26] Muehlschlegel S Selb J Patel M Diamond SG Franceschini MA Sorensen AG. Feasibility of NIRS in the neurointensive care unit: a pilot study in stroke using physiological oscillations. Neurocrit Care. (2009) 11:288–95. 10.1007/s12028-009-9254-4 19649749 PMC2782535

[27] Pennekamp CWA Immink RV Den Ruijter HM Kappelle LJ Ferrier CM Bots ML. Near-infrared spectroscopy can predict the onset of cerebral hyperperfusion syndrome after carotid endarterectomy. Cerebrovasc Dis. (2012) 34:314–21. 10.1159/000343229 23146912

[28] Shang Y Cheng R Dong L Ryan SJ Saha SP Yu G. Cerebral monitoring during carotid endarterectomy using near-infrared diffuse optical spectroscopies and electroencephalogram. Phys Med Biol. (2011) 56:3015–32. 10.1088/0031-9155/56/10/008 21508444

[29] Green MS Sehgal S Tariq R. Near-infrared spectroscopy: the new must have tool in the intensive care unit? Semin Cardiothorac Vasc Anesth. (2016) 20:213–24. 10.1177/1089253216644346 27206637

[30] Saito H Ishikawa T Tanabe J Kobayashi S Moroi J. Bedside assessment of regional cerebral perfusion using near-infrared spectroscopy and indocyanine green in patients with atherosclerotic occlusive disease. Sci Rep. (2018) 8:1242. 10.1038/s41598-018-19668-5 29352217 PMC5775286

[31] Selb J Wu K-C Sutin J Lin P-Y (Ivy) Farzam P Bechek S. Prolonged monitoring of cerebral blood flow and autoregulation with diffuse correlation spectroscopy in neurocritical care patients. Neurophotonics. (2018) 5:1. 10.1117/1.NPh.5.4.045005 30450363 PMC6233866

[32] Durduran T Choe R Baker WB Yodh AG. Diffuse optics for tissue monitoring and tomography. Reports Prog Phys. (2010) 73:076701. 10.1088/0034-4885/73/7/076701 26120204 PMC4482362

[33] Durduran T Yodh AG. Diffuse correlation spectroscopy for non-invasive, micro-vascular cerebral blood flow measurement. Neuroimage. (2014) 85:5163. 10.1016/j.neuroimage.2013.06.017 23770408 PMC3991554

[34] Ferradal SL Yuki K Vyas R Ha CG Yi F Stopp C. Non-invasive assessment of cerebral blood flow and oxygen metabolism in neonates during hypothermic cardiopulmonary bypass: feasibility and clinical implications. Sci Rep. (2017) 7:44117. 10.1038/srep44117 28276534 PMC5343476

[35] Shang Y Li T Yu G. Clinical applications of near-infrared diffuse correlation spectroscopy and tomography for tissue blood flow monitoring and imaging. Physiol Meas. (2017) 38:R1–26. 10.1088/1361-6579/aa60b7 28199219 PMC5726862

[36] Fantini S Sassaroli A Tgavalekos KT Kornbluth J. Cerebral blood flow and autoregulation: current measurement techniques and prospects for noninvasive optical methods. Neurophotonics. (2016) 3:031411. 10.1117/1.NPh.3.3.031411 27403447 PMC4914489

[37] Mesquita RC Durduran T Yu G Buckley EM Kim MN Zhou C. Direct measurement of tissue blood flow and metabolism with diffuse optics. Philos Trans R Soc A Math Phys Eng Sci. (2011) 369:4390–406. 10.1098/rsta.2011.0232 22006897 PMC3263785

[38] Selb J Boas DA Chan S-T Evans KC Buckley EM Carp SA. Sensitivity of near-infrared spectroscopy and diffuse correlation spectroscopy to brain hemodynamics: simulations and experimental findings during hypercapnia. Neurophotonics. (2014) 1:015005. 10.1117/1.NPh.1.1.015005 25453036 PMC4247161

[39] Culver JP Durduran T Furuya D Cheung C Greenberg JH Yodh AG. Diffuse optical tomography of cerebral blood flow, oxygenation, and metabolism in rat during focal ischemia. J Cereb Blood Flow Metab. (2003) 23:911–24. 10.1097/01.WCB.0000076703.71231.BB 12902835

[40] Valabrègue R Aubert A Burger J Bittoun J Costalat R. Relation between cerebral blood flow and metabolism explained by a model of oxygen exchange. J Cereb Blood Flow Metab. (2003) 23:536–45. 10.1097/01.WCB.0000055178.31872.38 12771568

[41] Hametner C Stanarcevic P Stampfl S Rohde S Veltkamp R Bösel J. Noninvasive cerebral oximetry during endovascular therapy for acute ischemic stroke: an observational study. J Cereb Blood Flow Metab. (2015) 35:1722–8. 10.1038/jcbfm.2015.181 26243709 PMC4635248

[42] Greisen G Leung T Wolf M. Has the time come to use near-infrared spectroscopy as a routine clinical tool in preterm infants undergoing intensive care? Philos Trans R Soc A Math Phys Eng Sci. (2011) 369:4440–51. 10.1098/rsta.2011.0261 22006900 PMC3263787

[43] Wolf M Ferrari M Quaresma V. Progress of near-infrared spectroscopy and topography for brain and muscle clinical applications. J Biomed Opt. (2007) 12:062104. 10.1117/1.2804899 18163807

[44] Proenca M Grossenbacher O Dasen S Moser V Ostojic D Lemkaddem A. Performance assessment of a dedicated reflectance pulse oximeter in a neonatal intensive care unit. In: 2018 40th Annual International Conference of the IEEE Engineering in Medicine and Biology Society (EMBC) IEEE (2018). pp. 1502–5. 10.1109/EMBC.2018.8512504 30440677

[45] Davies DJ Clancy M Dehghani H Lucas SJE Forcione M Yakoub KM. Cerebral oxygenation in traumatic brain injury: can a non-invasive frequency domain near-infrared spectroscopy device detect changes in brain tissue oxygen tension as well as the established invasive monitor? J Neurotrauma. (2019) 36:1175–83. 10.1089/neu.2018.5667 29877139

[46] Gagnon L Cooper RJ Yücel MA Perdue KL Greve DN Boas DA. Short separation channel location impacts the performance of short channel regression in NIRS. Neuroimage. (2012) 59:2518–28. 10.1016/j.neuroimage.2011.08.095 21945793 PMC3254723

[47] Gagnon L Perdue K Greve DN Goldenholz D Kaskhedikar G Boas DA. Improved recovery of the hemodynamic response in diffuse optical imaging using short optode separations and state-space modeling. Neuroimage. (2011) 56:1362–71. 10.1016/j.neuroimage.2011.03.001 21385616 PMC3085546

[48] Goodwin JR Gaudet CR Berger AJ. Short-channel functional near-infrared spectroscopy regressions improve when source-detector separation is reduced. Neurophotonics. (2014) 1:015002. 10.1117/1.NPh.1.1.015002 26157972 PMC4478749

[49] Huppert TJ. Commentary on the statistical properties of noise and its implication on general linear models in functional near-infrared spectroscopy. Neurophotonics. (2016) 3:010401. 10.1117/1.NPh.3.1.010401 26989756 PMC4773699

[50] Kirilina E Jelzow A Heine A Niessing M Wabnitz H Brühl R. The physiological origin of task-evoked systemic artefacts in functional near infrared spectroscopy. Neuroimage. (2012) 61:70–81. 10.1016/j.neuroimage.2012.02.074 22426347 PMC3348501

[51] Yücel MA Selb J Aasted CM Lin P-Y Borsook D Becerra L. Mayer waves reduce the accuracy of estimated hemodynamic response functions in functional near-infrared spectroscopy. Biomed Opt Express. (2016) 7:3078. 10.1364/BOE.7.003078 27570699 PMC4986815

[52] Farzam P Buckley EM Lin P-Y Hagan K Grant PE Inder TE. Shedding light on the neonatal brain: probing cerebral hemodynamics by diffuse optical spectroscopic methods. Sci Rep. (2017) 7:15786. 10.1038/s41598-017-15995-1 29150648 PMC5693925

[53] Busch DR Rusin CG Miller-Hance W Kibler K Baker WB Heinle JS. Continuous cerebral hemodynamic measurement during deep hypothermic circulatory arrest. Biomed Opt Express. (2016) 7:3461. 10.1364/BOE.7.003461 27699112 PMC5030024

[54] Wong F Cerebral blood flow measurements in the neonatal brain. In: Prenatal and Postnatal Determinants of Development. New York, NY: Humana Press (2016). pp. 69–87. 10.1007/978-1-4939-3014-2_5

[55] Jain V Buckley EM Licht DJ Lynch JM Schwab PJ Naim MY. Cerebral oxygen metabolism in neonates with congenital heart disease quantified by MRI and optics. J Cereb Blood Flow Metab. (2014) 34:380–8. 10.1038/jcbfm.2013.214 24326385 PMC3948119

[56] Favilla CG Mesquita RC Mullen M Durduran T Lu X Kim MN Optical bedside monitoring of cerebral blood flow in acute ischemic stroke patients during head-of-bed manipulation. Stroke. (2014) 45:1269–74. 10.1161/STROKEAHA.113.004116 24652308 PMC4006296

[57] Yu G Diffuse correlation spectroscopy (DCS): a diagnostic tool for assessing tissue blood flow in vascular-related diseases and therapies. Curr Med Imaging Rev. (2012) 8:194–210. 10.2174/157340512803759875

[58] Buckley EM Lynch JM Goff DA Schwab PJ Baker WB Durduran T. Early postoperative changes in cerebral oxygen metabolism following neonatal cardiac surgery: effects of surgical duration. J Thorac Cardiovasc Surg. (2013) 145:196–205.e1. 10.1016/j.jtcvs.2012.09.057 23111021 PMC3658109

[59] Oliveira ML de Azevedo D de Azevedo M Nogueira RC Teixeira M Bor-Seng-Shu E Encephalic hemodynamic phases in subarachnoid hemorrhage: how to improve the protective effect in patient prognoses. Neural Regen Res. (2015) 10:748 10.4103/1673-5374.156969 26109948 PMC4468765

[60] Martin NA Patwardhan RV Alexander MJ Africk CZ Lee JH Shalmon E. Characterization of cerebral hemodynamic phases following severe head trauma: hypoperfusion, hyperemia, and vasospasm. J Neurosurg. (1997) 87:9–19. 10.3171/jns.1997.87.1.0009 9202259

[61] Carpenter DA Grubb RL Tempel LW Powers WJ. Cerebral oxygen metabolism after aneurysmal subarachnoid hemorrhage. J Cereb Blood Flow Metab. (1991) 11:837–44. 10.1038/jcbfm.1991.143 1874816

[62] Yundt KD Grubb RL Diringer MN Powers WJ. Autoregulatory vasodilation of parenchymal vessels is impaired during cerebral vasospasm. J Cereb Blood Flow Metab. (1998) 18:419–24. 10.1097/00004647-199804000-00010 9538907

[63] Oddo M Levine JM Frangos S Maloney-Wilensky E Carrera E Daniel RT. Brain lactate metabolism in humans with subarachnoid hemorrhage. Stroke. (2012) 43:1418–21. 10.1161/STROKEAHA.111.648568 22343642

[64] Yokose N Sakatani K Murata Y Awano T Igarashi T Nakamura S. Bedside monitoring of cerebral blood oxygenation and hemodynamics after aneurysmal subarachnoid hemorrhage by quantitative time-resolved near-infrared spectroscopy. World Neurosurg. (2010) 73:508–13. 10.1016/j.wneu.2010.02.061 20920934

[65] de Lima Oliveira M Kairalla AC Fonoff ET Martinez RCR Teixeira MJ Bor-Seng-Shu E. Cerebral microdialysis in traumatic brain injury and subarachnoid hemorrhage: state of the art. Neurocrit Care. (2013) 21:152–62. 10.1007/s12028-013-9884-4 24072457

[66] Gagnon L Desjardins M Jehanne-Lacasse J Bherer L Lesage F. Investigation of diffuse correlation spectroscopy in multi-layered media including the human head. Opt Express. (2008) 16:15514. 10.1364/OE.16.015514 18825190

[67] Liemert A Kienle A. Light diffusion in a turbid cylinder II Layered case. Opt Express. (2010) 18:9266. 10.1364/OE.18.009266 20588774

[68] Hallacoglu B Sassaroli A Fantini S. Optical characterization of two-layered turbid media for non-invasive, absolute oximetry in cerebral and extracerebral tissue. PLoS ONE. (2013) 8:e64095. 10.1371/journal.pone.0064095 23724023 PMC3660388

[69] Alexandrakis G Busch DR Faris GW Patterson MS. Determination of the optical properties of two-layer turbid media by use of a frequency-domain hybrid Monte Carlo diffusion model. Appl Opt. (2001) 40:3810. 10.1364/AO.40.003810 18360415

[70] Baker WB Parthasarathy AB Ko TS Busch DR Abramson K Tzeng S-Y. Pressure modulation algorithm to separate cerebral hemodynamic signals from extracerebral artifacts. Neurophotonics. (2015) 2:035004. 10.1117/1.NPh.2.3.035004 26301255 PMC4524732

[71] Verdecchia K Diop M Lee A Morrison LB Lee T-Y St. Lawrence K. Assessment of a multi-layered diffuse correlation spectroscopy method for monitoring cerebral blood flow in adults. Biomed Opt Express. (2016) 7:3659. 10.1364/BOE.7.003659 27699127 PMC5030039

[72] He L Baker WB Busch DR Jiang JY Lawrence KS Kofke WA. Noninvasive continuous optical monitoring of absolute cerebral blood flow in critically ill adults. Neurophotonics. (2018) 5:1. 10.1117/1.NPh.5.4.045006 30480039 PMC6251207

[73] Diop M Verdecchia K Lee T-Y St. Lawrence K. Calibration of diffuse correlation spectroscopy with a time-resolved near-infrared technique to yield absolute cerebral blood flow measurements. Biomed Opt Express. (2011) 2:2068. 10.1364/BOE.2.002068 21750781 PMC3130590

[74] Milej D He L Abdalmalak A Baker WB Anazodo UC Diop M. Quantification of cerebral blood flow in adults by contrast-enhanced near-infrared spectroscopy: validation against MRI. J Cereb Blood Flow Metab. (2019). 10.1177/0271678X19872564. [Epub ahead of print]. 31500522 PMC7370369

