# Supplementary material for: Real-Time Non-invasive Assessment of Cerebral Hemodynamics With Diffuse Optical Spectroscopies in a Neuro Intensive Care Unit: An Observational Case Study
Source: Front Med (Lausanne). 2020 Apr 28;7:147. doi: 10.3389/fmed.2020.00147 (PMC7198738; doi:10.3389/fmed.2020.00147)
Supplement: Supplementary file 1 [file Data_Sheet_1.PDF]

Supplementary Material

1 Supplementary methods

1.1 Optical instrumentation

For this study, we developed a hybrid diffuse optical instrument employing both a DCS module and a commercial FD-DOS module (Supplementary Figure 1-A). The homemade DCS instrument employs 16 single photon-counting detector arrays (SPCM-AQ4C, Pacer, USA) and one long-coherence laser emitting at 785 nm (DL785-100-SO, CrystaLaser, USA). The signal from the detector feeds a hardware correlator (Correlator.com, China) that outputs the DCS auto-correlation curves. The FD-DOS module (Imagent, ISS Inc., USA) employs 4 photomultiplier tubes as detectors and 32 laser diodes modulated at 110 MHz that provide 8 sources, with four different wavelengths each (690, 705, 750 and 846 nm).

To permit FD-DOS and DCS measurements, we used an Arduino board programmed inside our control software to automatically switch between each module. The interleaved measurements of FD-DOS and DCS is necessary in order to avoid damaging the PMTs of the FD-DOS module due to the high power of the DCS source. The total sampling rate of our system is ~0.16 Hz. We developed a user-friendly graphical user interface (GUI) with LabVIEW (National Instruments, USA) to control the operation of both modules and to display in *real-time* CBF, OEF, and $CMRO_2$. The GUI was based on current instruments available at the neuro-ICU, and it was adapted after feedback by clinical users in order to facilitate its use by hospital personnel, such as neurointensivists and nurses.

The optical probe was a small rubber pad of approximately $2 \times 5cm$ and consisted of one DCS source and three DCS detectors combined with four DOS sources and one DOS detector, providing a total of three DCS source-detector combinations (separated at 1.5, 2.0, and 2.5 cm), and four DOS source-detector combinations (separated at 1.5, 2.0, 2.5 and 3.0 cm) (Supplementary Figure 1-B). In this work, we opted to focus on a single DCS source-detector separation (2.5 cm) and only two DOS wavelengths (690 and 846 nm). With these separations, we were capable of probing the most external surface of the prefrontal cortex (PFC), as it was shown in previous studies (1,2).

1.2 Optical analysis

We employed a multi-spectral fitting algorithm to analyze the FD-DOS data. Briefly, we fit the measured light intensity (AC_{Meas}) and phase (Ph_{Meas}) to the theoretical light intensity (AC_{Theo}) and phase (Ph_{Theo}) extracted from the solution of the diffusion equation for the light fluence rate in turbid media assuming a semi-infinite homogeneous medium (3). In our approach, all DOS source-detector separations at all wavelengths were fit simultaneously to recover the HbO and HbR concentrations as well as the scattering parameters A and b. In this model, the reduced scattering coefficient is given by $\mu_s' = A(\lambda/800)^{-b}$, where λ is the wavelength (in nm) and A and b are related to the density and shape of the scatterers in tissue (3,4). Similarly, the absorption coefficient, μ_a, is given by:

$$\mu_a = \varepsilon_{HbO} HbO + \varepsilon_{HbR} HbR + f_{H_2O} \mu_{a_{H_2O}},$$

where ε_{HbO} and ε_{HbR} are extinction coefficients of oxy- and deoxy-hemoglobin, and f_{H_2O} is the water concentration fraction, assumed to be 0.75. We used a Nelder-Mead non-linear least squares algorithm to minimize the cost-function:

$$\chi^2 = \chi^2_{Ph} + \chi^2_{AC},$$

where

$$\chi^2_{AC} = \sum_{\lambda} \sum_{\rho_1} \sum_{\substack{\rho_2 \\ \rho_1 \neq \rho_2}} \left[log \frac{AC_{Meas}(\rho_1,\lambda)}{AC_{Meas}(\rho_2,\lambda)} - log \frac{AC_{Theo}(\rho_1,\lambda)}{AC_{Theo}(\rho_2,\lambda)} \right]^2$$

and

$$\chi^2_{Ph} = \sum_{\lambda} \sum_{\rho_1} \sum_{\substack{\rho_2 \\ \rho_1 \neq \rho_2}} \left[\left(Ph_{Meas}(\rho_1,\lambda) - Ph_{Meas}(\rho_2,\lambda) \right) - \left(Ph_{Tho}(\rho_1,\lambda) - Ph_{Theo}(\rho_2,\lambda) \right) \right]^2.$$

To improve the stability of our fitting procedure, we first calculated the average scattering coefficients (i.e., *A* and *b*) from either the initial or the last 10 minutes of data (depending on the hemisphere placement order). Then, using the fixed parameters for *A* and *b,* we computed the HbO and HbR concentrations from each time point.

For the DCS analysis, we first estimated the absorption and scattering coefficients at the DCS wavelength (i.e., 785 nm) using the parameters recovered from the multi-spectral fit of our DOS data. Then, we fit the measured autocorrelation function to a semi-infinite homogeneous dynamic model to recover a cerebral blood flow (CBF) index (3,5). For the DCS data, each source-detector separation was fit independently. All analysis scripts were written using open-source libraries based on Python 3 (6–8).

1.3 References

1. Kim MN, Durduran T, Frangos S, Edlow BL, Buckley EM, Moss HE, Zhou C, Yu G, Choe R, Maloney-Wilensky E, et al. Noninvasive Measurement of Cerebral Blood Flow and Blood Oxygenation Using Near-Infrared and Diffuse Correlation Spectroscopies in Critically Brain-Injured Adults. *Neurocrit Care* (2010) **12**:173–180. doi:10.1007/s12028-009-9305-x

2. Selb J, Boas DA, Chan S-T, Evans KC, Buckley EM, Carp SA. Sensitivity of near-infrared spectroscopy and diffuse correlation spectroscopy to brain hemodynamics: simulations and experimental findings during hypercapnia. *Neurophotonics* (2014) **1**:015005. doi:10.1117/1.NPh.1.1.015005

3. Durduran T, Choe R, Baker WB, Yodh AG. Diffuse optics for tissue monitoring and tomography. *Reports Prog Phys* (2010) **73**:076701. doi:10.1088/0034-4885/73/7/076701

4. Jacques SL. Optical properties of biological tissues: a review. *Phys Med Biol* (2013) **58**:R37–R61. doi:10.1088/0031-9155/58/11/R37

5. Durduran T, Yodh AG. Diffuse correlation spectroscopy for non-invasive, micro-vascular

cerebral blood flow measurement. *Neuroimage* (2014) **85**:5163. doi:10.1016/j.neuroimage.2013.06.017

6. Newville M, Stensitzki T, Allen DB, Ingargiola A. LMFIT: Non-Linear Least-Square Minimization and Curve-Fitting for Python. *Zenodo* (2014) doi:10.5281/zenodo.11813

7. Oliphant TE. SciPy: Open source scientific tools for Python. *Comput Sci Eng* (2007) doi:10.1109/MCSE.2007.58

8. Hunter JD. Matplotlib: A 2D graphics environment. *Comput Sci Eng* (2007) doi:10.1109/MCSE.2007.55

2 Supplementary Figures

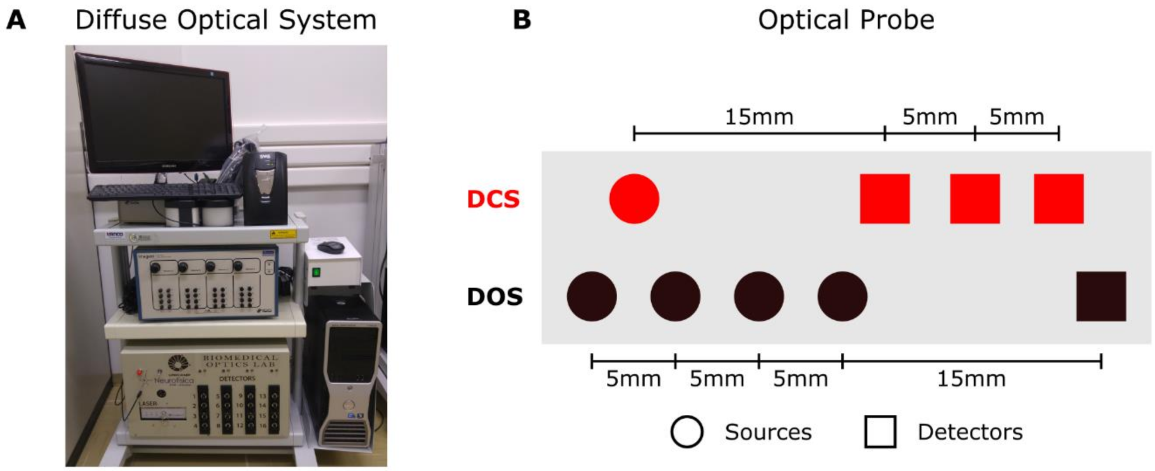

Supplemental Figure 1. Instrumentation utilized for this study. **(a)** The diffuse optical system, which combines a frequency-domain diffuse optical spectroscopy (DOS) module and a diffuse correlation spectroscopy (DCS) module. **(b)** A schematic of the optical probe employed in this study, which consisted of three DCS source-detector separations (1.5, 2.0 and 2.5 cm, in red) and four DOS source-detector separations (1.5, 2.0, 2.5 and 3.0 cm, in black).

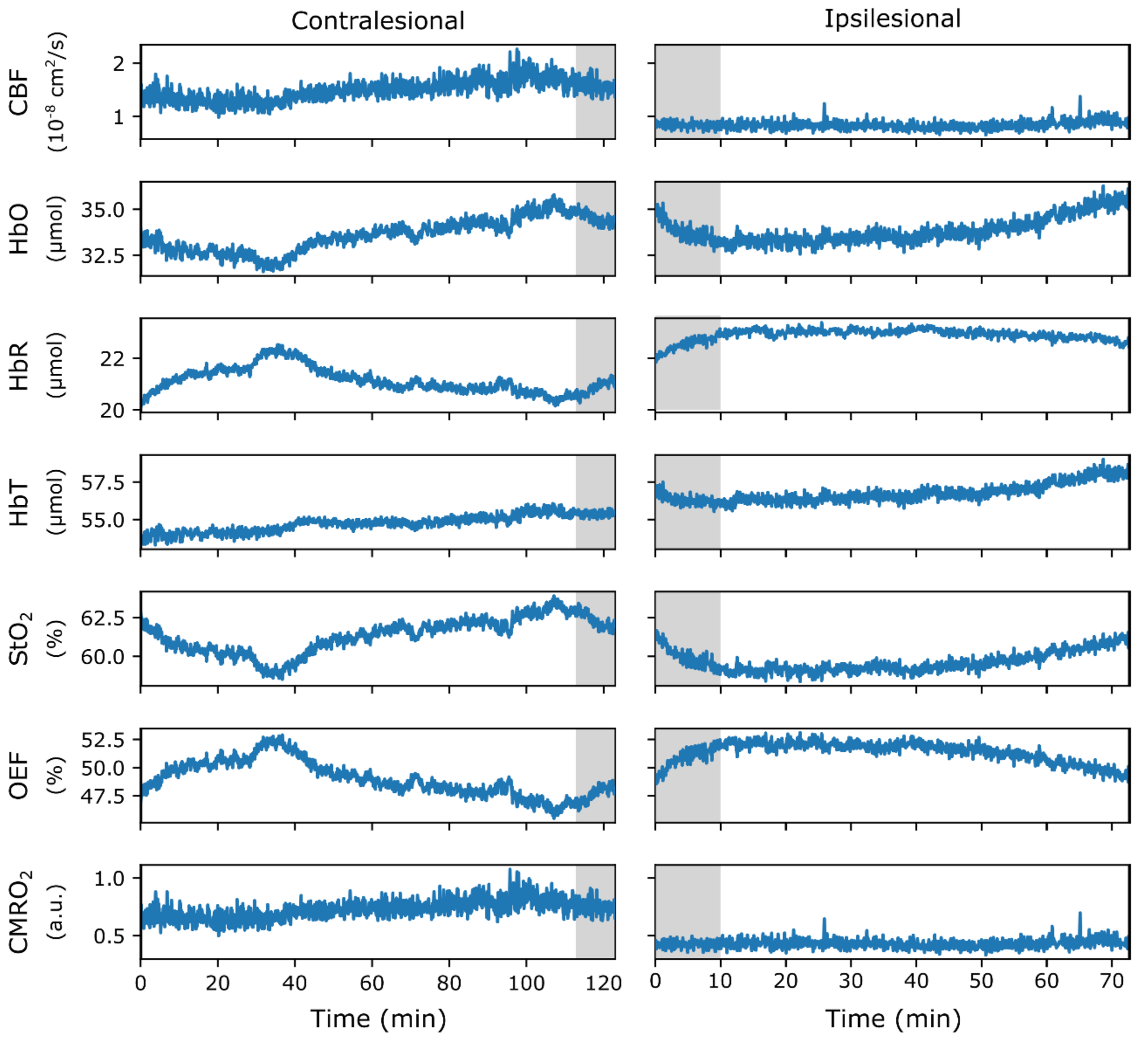

Supplementary Figure 2 - All optical derived parameters, day-by-day. The gray-shaded area represents the initial and final 10 minutes used for averaging. In this case, the contralesional hemisphere was measured before the ipsilesional hemisphere. (CBF: cerebral blood flow; HbO: oxy-hemoglobin concentration; HbR: deoxy-hemoglobin concentration; HbT: total hemoglobin concentration; St: oxygen saturation; OEF: oxygen extraction fraction; $CMRO_2$: cerebral metabolic rate of oxygen).
